# Formulation and Development of a Validated UV-Spectrophotometric Analytical Method of Rutin Tablet

**DOI:** 10.1155/2017/2624947

**Published:** 2017-05-16

**Authors:** Murad N. Abualhasan, Jumana Mansour, Nidal Jaradat, Abdel Naser Zaid, Ibrahim Khadra

**Affiliations:** ^1^Department of Pharmacy, Faculty of Medicine & Health Sciences, An-Najah National University, P.O. Box 7, Nablus, State of Palestine; ^2^Strathclyde Institute of Pharmacy and Biomedical Sciences, University of Strathclyde, Glasgow, UK

## Abstract

Rutin is available in some foods, fruits, and vegetables. It has various beneficial medical effects making it useful in the treatment of various diseases. Rutin is available in different oral dosage forms such as tablets or capsules, widely available in the market. Rutin and many herbal medicines lack quality control due to unavailability of analytical methods. In this study, we formulated rutin tablet and studied its stability using a simple developed analytical method. The dissolution profile of our formulated tablet was also inspected. The results showed that our developed method was linear (*R*^2^ = 0.999), precise (% RSD = 0.026), and accurate (% recovery = 98.55–103.34). The formulated rutin tablet was stable under accelerated conditions as well as room temperature for 150 days (% assay > 91.69). The dissolution profile over 45 minutes of our formulated tablet showed a better dissolution (26.5%) compared with the internationally marketed Rutin® tablet (18.5%). This study can serve as a guideline to companies that manufacture herbal products to improve their formulated herbs and apply validated analytical methods to check the quality of their product.

## 1. Introduction

**Figure 1:**
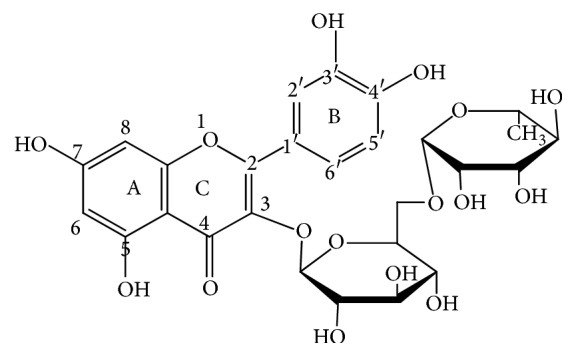
Chemical structure of rutin.

Rutin is 3,3′,4′,5,7-pentahydroxy flavones-3-rutinoside. It is the yellow crystalline rhamnoglucoside of the flavonoid quercetin and has a chemical formula C_27_H_30_O_16_ with chemical structure as shown in Figure 1.

Rutin is slightly soluble in water and has a higher solubility in organic solvent such as methanol. It showed low bioavailability after studying it in animals and human volunteers, due to its low water solubility [1].

Rutin is found in some foods, fruits, vegetables, and plant-based beverages such as buckwheat, onions, apples, berries, orange, grape fruit, lemon, tea, and asparagus. Rutin has various beneficial medical effects: it possesses antioxidant and anti-inflammatory effects and is widely used for hemorrhage and varicose [2, 3].

Various dosage forms are available in the local and international market such as tablets and capsules and in topical applications such as gels. Rutin is available in these dosage forms either alone or in combination with other active ingredients. Liquid chromatography and UV spectrophotometer methods are the most popular methods of rutin analysis [4, 5]. Tablets are the mostly used dosage forms available in the market. Formulations of tablets include diluents, binders, and disintegrants. The selection of these excipients should take into consideration the physical and chemical properties which include compatibility, flowability, solubility, hygroscopicity, lubricity, and its effect on stability of the tablet [6–10].

Employment of a fully validated analytical method is highly needed to quantify rutin in various dosages. The typical validation parameters should be considered in the analytical validation procedure; these parameters include accuracy, precision, repeatability, intermediate precision, specificity, detection limit, quantitation limit, linearity, and range [11–15]. The ICH guidelines clearly stated the requirement and establishment of stability-indicating assay method. It requires the conduct of forced decomposition studies under a variety of conditions, like pH, light, oxidation, and dry heat. The drug must be separated from the degradation products and the method must be able to analyze each individual degradation product [16, 17].

To our knowledge, there is no pharmacopeial method or any validated method that quantifies rutin in its final dosage form. The objective of this study is to formulate a rutin tablet and compare its quality (mainly tablet dissolution) with what is available in the local and international market using a home developed method. The method will be validated according to the international standards [18, 19]. The developed analytical method will be applied in quantification of rutin in both raw material and its final tablet dosage form. The dissolution of our developed tablet formulation will be evaluated and will be compared to the dissolution of other rutin tablets that are available in the market. Moreover, a stability study under normal and stress conditions will be conducted for our formulated tablet [20]. Our validated methods can be included in one of the international pharmacopeia. Moreover, the quality R&D and quality control lab sections of the herbal industry can benefit from this research project to improve their herbal products and control its quality.

## 2. Materials and Methods

### 2.1. Materials

All reagents used in this study had the minimum requirements set by American Society for Testing Material (ASTM) and American Chemical Society (ACS) specifications for analytical reagents. All the chemicals used were purchased from reliable sources; these chemicals and materials include the following: acetone, acetonitrile, ethanol, and potassium dihydrogen phosphate were purchased from Sigma Aldrich, St. Louis, USA. Hydrochloric acid 32%, hydrogen peroxide 30%, isopropyl alcohol, methanol, and tetrahydrofuran were purchased from Thermo Fisher GmbH, Karlsruhe, Germany.

Rutin trihydrate powder 99% was purchased from Sigma Aldrich, St. Louis, USA. All tablet excipients were given as a gift from the Jerusalem Pharmaceutical Company, and these excipients include aerosil, acdisol, magnesium stearate, and microcrystalline cellulose (MCC). Rutin tablet (Solgar-Leonia, New Jersey, USA) was purchased from a local community pharmacy shop and was used as a market reference.

### 2.2. Instrumentation

The instruments that were used during our research include the following: disintegration testers (Erweka, Model-ZT 220, Germany), UV/visible spectrophotometer (JENWAY, Model-7315, Staffordshire, UK), Paddle Dissolution Tester (HSIANGTAI, Model-DT-6), pH meter (JENWAY, Model-3510 Staffordshire, UK), hotplate stirrer (LabTech Model-ES35A, Hopkinton, USA), analytical balance (Nevada Weighing, Model-220), and multicheck of hardness, thickness, and diameter (Erweka, Model-5.1, Germany), oven (BINDER, Model-ED56, Germany), and rotavapor (Heidolph, Model-VV2000, Schwabach, Germany).

### 2.3. Analytical Method Development

#### 2.3.1. Determination of Wavelength of Maximum Absorption (*λ*max) of Rutin

Rutin (0.1 mg/ml) was dissolved in solvent diluent (methanol : water; 9 : 1) and the UV-Vis spectrum of rutin trihydrate was tested using UV spectrophotometer in the range of 200–800 nm. The interfering effect of excipients on the maximum absorption (*λ*max) was tested by scanning the spectrum of each excipient alone as well as in combination with rutin.

#### 2.3.2. Determination of Rutin Hydrochloride in Bulk

The developed method was applied to determine rutin in bulk. An accurate weight (10 mg) of rutin bulk material was transferred into a 100 ml volumetric flask containing 20 ml of diluent and the volume was made up to the mark using the same diluent. Appropriate volume 40 ml of this solution was transferred to a 100 ml volumetric flask, and the volume was adjusted to the mark using diluent. The absorption was recorded at 360 nm and the concentrations of the drug were calculated from linear regression equations. The % recovery and % RSD of the rutin in bulk were calculated for 5 repeated tests.

#### 2.3.3. Analysis of Formulated and Commercial Tablet Formulation

The developed method was also applied to determine rutin in tablets. An average weight of rutin tablet was taken in a 250 ml volumetric flask and the volume was made up to the mark with diluent. From this, 4 ml was taken and transferred to a 100 ml volumetric flask and the volume was made up to the mark with diluent to give 0.04 mg/ml concentration. The absorption was recorded at 360 nm and the concentrations of the drug were calculated from the linear regression equation. The % assay and % RSD of the rutin in tablets were calculated for 5 repeated tests.

### 2.4. Analytical Method Validation

The linearity and range of the developed method were performed by measuring the absorption of a series of rutin standard solutions (0.009–0.09 mg/ml) at *λ*_max_ of 360 nm. The absorptions of these standard solutions were plotted against their concentration. The regression line equation and the square of the correlation coefficient (*R*^2^) were calculated.

The accuracy and precision validation parameters were evaluated by testing three concentrations (80%, 100%, and 120%) of the rutin theoretical value, and three replicates of each concentration were tested.

The recovery and precision were performed by testing three prepared working solutions that are equivalent to 80, 100, and 125% of the test concentration. Triplicate measurements were done for each prepared solution. The measurements were repeated for three consecutive days. The percentage recovery and % RSD were then calculated.

The selectivity of the method was carried out by measuring the absorbance of the excipients mixture without the active ingredient. The absorbance was measured in the range of 200–800 nm. The resulting spectrum of the excipients was compared to that of rutin and was checked for any interference at measuring *λ*_max_ of 360 nm. The method specificity and selectivity were also checked for any interference of degradative substances. This was performed by subjecting the sample solution to forced degradation conditions. Forced degradation was carried out by exposing the formulation solution (0.04 mg/ml) to four stress conditions, 0.1 N HCl, 0.1 N NaOH, 0.3% H_2_O_2_, and UV light at 254 nm for 3 hrs. Moreover, the rutin formulated tablets were subjected to high temperature (40°C) for 150 days.

The robustness of the method was performed by examining the effect of slight changes on absorption at wavelengths 360 ± 2, the effect of slight changes in diluents composition in the ratio of methanol : water; 9 : 2, and methanol : water; 10 : 1, was also examined. The effect of changing personnel has been studied by using another analyst.

The LOD and LOQ of the method were calculated based on the standard deviation of the response (*σ*) and slope approach as defined in ICH guidelines [21]. LOD and LOQ were calculated according to 3.3*∗σ*/Slope for LOD and 10*∗σ*/Slope for LOQ.

### 2.5. Tablet Formulation Development

**Figure 2:**
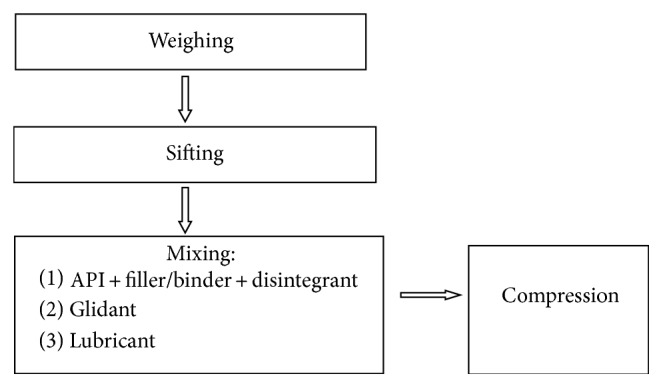
Steps of formulation preparation.

Three different formulae of Rutin 250 mg tablets were prepared in our research lab. The components used are rutin trihydrate, magnesium stearate, microcrystalline cellulose (MCC), aerosil, and acdisol. The detailed composition of the three tablet formulations are listed in Table 1. The tablets were prepared by “direct compression” method [9], according to the flowchart shown in Figure 2.

### 2.6. Dissolution Profile of Formulated Rutin Tablet

Dissolution was done according to USP and ICH guidelines [22, 23]. The dissolution was done using USP apparatus 2 (paddle). The dissolution test was performed in three different pH media of 1.2 (0.1 N HCl), 4.5, and 6.8 phosphate buffer prepared according to USP. The dissolution apparatus was run at 50 rpm and 37°C for 45 minutes. One tablet was placed into each of the six dissolution vessels containing 900 ml of dissolution medium. 10 ml of the sample was withdrawn by syringes from each dissolution vessel at time intervals of 5, 10, 15, 20, 25, 30, 35, 40, and 45 minutes. The average reading of triplicate measurements was taken to calculate the percentage of dissolved rutin using the following formula:

(1) %  of  dissolved  Rutin=Actual  amount  of  released  rutinTheoretical  amount  of  rutin  in  tablet∗100.

The dissolution profiles of the formulated tablets and the marketed rutin tablet were studied in the selected dissolution media. Comparison of dissolution was based on the values of the calculated similarity factor (*f*_2_) and dissimilarity factor (*f*_1_) of the dissolution profile for the formulated and marketed rutin tablets. Values for *f*_1_ and *f*_2_ were calculated using (2) and (3), respectively. The *f*_2_ factor measures the closeness between two profiles and *f*_1_ measures difference between two profiles. *R*_*t*_ and *T*_*t*_ in equations represent the percentages of drug dissolved at each time point for the reference (market tablet) and test (formulated tablet), respectively. An *f*_1_ value greater than 15 indicates significant dissimilarity, and an *f*_2_ value greater than 50 indicates significant similarity [24–26]:

(2) f1=∑t=1nRt−Tt∑t=1nRt×100,

(3) f2=50·log⁡1+1n∑t=1nRt−Tt2−0.5×100.

### 2.7. Weight Variation and Content Uniformity of the Formulated Tablets

Weight variation of the formulated tablets was performed in accordance with the USP method specified for uncoated tablets [19].

The weight variation is done by weighing 20 tablets individually and the test will be considered successful if it meets the requirements set by the official pharmacopeia.

The content uniformity test was done in accordance with USP. The rutin content for each tablet was calculated relative to the label claim.

### 2.8. The Physical Specification

The disintegration time of our formulated tablet was performed according to USP [27]. When all the tablets have been completely disintegrated, this was recorded as disintegration time.

Tablet physical specifications like the hardness, thickness, and diameter were determined and tested using multicheck* (Erweka 5.1)*. The test was done on ten tablets; the average reading was assigned as the tablet hardness, thickness, and diameter specifications of the formulated tablets.

### 2.9. Stability of Formulated Rutin Tablet

The stability of the formulated rutin tablet was studied by storing the tablet at room temperature as well as at 40°C and analyzed periodically by using the developed analytical test method. The percentage content of formulated rutin tablets was calculated periodically through 150 days.

## 3. Results

### 3.1. Analytical Method Development

**Figure 3:**
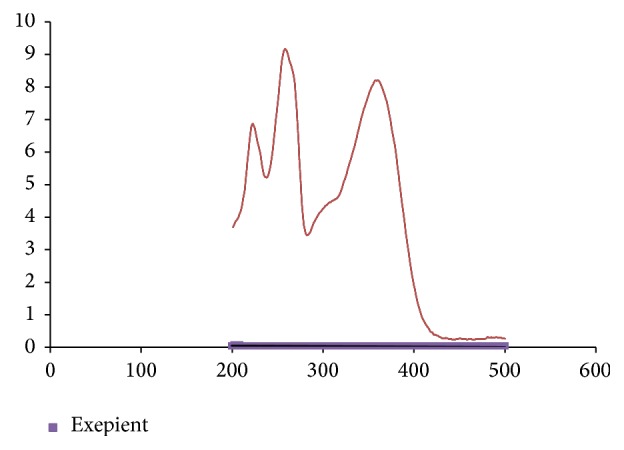
Spectrum of rutin and excipients in the range of 200–800 nm.

The spectrum of rutin solution in the range of 200–800 nm showed two absorption maxima, at 360 nm and at 260 nm. The results also demonstrated no interference of the excipient used in formulation at the measuring absorption maxima (Figure 3).

The developed method was applied to determine rutin in bulk and tablets. The % amount of bulk rutin recovered was between 98.92% and 100.33% and the % RDS was 1.22. The % assay of rutin tablets was between 98.24% and 101.31% and the RSD was 1.32.

### 3.2. Analytical Method Validation Results

The absorption of serial standard solutions in the range of 0.009–0.09 mg/ml was plotted against its concentration and the regression line was examined for linearity over the concentration range. The curve was linear with a regression line equation of *y* = 25.035*x* + 0.0634. The goodness-of-fit (*R*^2^) was also found to be 0.999 indicating a linear relationship in the mentioned range.

The accuracy and precision of the method were established on the results of three concentration levels around concentration test value of rutin (80%, 100%, and 120%). The results of the recovery of rutin active ingredient showed a good accuracy (98.55–103.34).

The prepared solutions were tested for precision in three replicates and an intraday testing for three consecutive days (intermediate precision). The results indicate that the method is precise; the % RSD for the intraday was in the acceptable range (0.016–0.026) and *P* value of the ANOVA results (intermediate precision) was >0.05 (Table 2).

The results stability-indicating study of formulated rutin tablet under stress conditions of 0.1 N NaOH, 0.1 N HCl, UV light (254 nm), and 0.3% H_2_O_2_ showed that rutin tablets are only stable in the UV light and slightly degraded in the H_2_O_2_ as the percentage assay has dropped from 104% to about 80%. The results also demonstrate that instant degradation has occurred on the tablet after the addition of alkaline and acidic solution, and the assay dropped to about 60%.

### 3.3. Weight Variation and Content Uniformity of Rutin Tablet

Weight variation was performed according to USP; the results show that the variation for any of tested tablets was not more than 2.6% from the mean weight.

The uniformity content test was also performed according to the USP; the results show that % RSD value of the assayed tablets was 1.05 and no tablet % assay was out of the limit (85–115%).

### 3.4. Dissolution Profile

**Figure 4:**
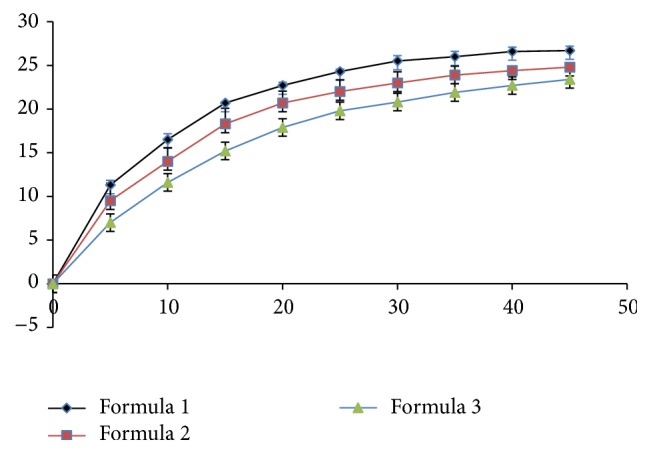
Dissolution profile of three formulations in the dissolution media (pH 6.8).

The dissolution profile was done to compare in vitro dissolution profiles of different rutin tablet formulation. The results clearly demonstrate that formula 1 (F1) has the best dissolution among three formulations. Thus, we selected formula 1 (F1) for the shelf life, accelerated, and stress stability studies (Figure 4).

The results also show a moderate dissolution (26.7%) after 45 minutes, but it reaches a plateau after approximately 20 minutes. The low dissolution of the tablet was due to the low solubility of rutin in aqueous media.

**Figure 5:**
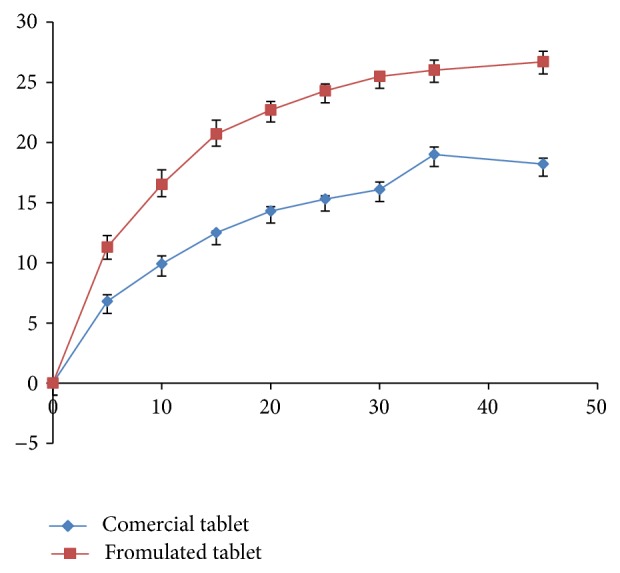
Dissolution profile for formulated rutin and commercial rutin tablet at phosphate buffer media (pH = 6.8).

In order to test the dissolution profile of the other dissolution media, F1 was tested in three different dissolution media, namely, phosphate buffer 6.8, phosphate buffer 4.5, and 0.1 N HCl; the dissolution was not significantly different between the three media (*P* < 0.05). Furthermore, the dissolution for the formulated rutin tablet (F1) and for marketed rutin tablet was tested using phosphate buffer of pH 6.8. The results (Figure 5) show slightly higher dissolution for our formulated tablet compared to the rutin tablet.

### 3.5. The Physical Specification

Some of the physical parameters of our formulated tablet including disintegration, hardness, thickness, and diameter were determined as tablet specification.

The disintegration of the tablet was performed using USP specified disintegration apparatus. The tablets were placed in the specified baskets and observed for complete disintegration. The formulated tablets were seen to disintegrate totally after 4 minutes.

The tablets were tested for their hardness, thickness, and diameter simultaneously using Erweka multicheck instrument. The average value of the tested parameters will be considered as our tablet specification.  Table 3 shows the detailed data of the tested parameters.

### 3.6. Stability of Formulated Rutin Tablet

To study the tablet's stability, the formulated tablets were stored in room temperature at 40°C and were analyzed periodically through 150 days. The results indicate the tablets and the tablet assay after 150 days at room temperature and 40°C were 96.33 and 91.69, respectively (Table 4).

## 4. Discussions

The spectrum shows two absorption maxima, at 360 nm and at 260 nm. The absorption at 260 nm was linear with an *R*2 almost similar to 360 nm. However, we adapted 360 nm as a measuring wavelength in our analytical method to avoid any absorption from the excipient and degradation product. To examine absorptivity of the excipients on the measuring *λ*_max_ 360 nm, all the expected excipients which were included in formulation were dissolved in the diluents and their absorbance was measured. The results show that the absorbance at the selected *λ*_max_ is negligible relative to rutin absorption at *λ*_max_ 360 nm. The result clearly demonstrates that there is no interaction between the excipients and rutin active ingredient and the wavelength is selective for rutin. The specificity and selectivity of the developed analytical method were also tested by stability-indicating study. The formulated rutin tablet was put under different stress conditions: 0.1 N NaOH, 0.1 N HCl, UV light (254 nm), and 0.3% H_2_O_2_. The results showed that method can selectively quantify any drop in the assay of rutin in the formulated tablets under the above selected stress conditions.

The results clearly demonstrate that our formulated tablets comply with weight variation and the content uniformity; according to USP, weight variation test will pass only if not more than two of the individual weights deviate from the average weight by ±7.5% and none deviates by more than twice that percentage. Our results show that the variation for any of the tested tablets was not more than 2.6% from the mean weight. The uniformity content according to the USP will pass if the relative standard deviation (% RSD) is ≤15 and no % assay value is outside 85–115%. The test fails if one or more values are outside 75–125%. The uniformity content test results showed that RSD value of the assayed tablets was 1.05% and no tablet has % assay that was out of the limit (85–115%). The rutin dissolution profile of the formulated tablet showed a slight dissolution improvement over the marketed rutin tablet. However, the result of similarity factor (f2) was >50 and the dissolution data revealed that there was no statistical difference (*P* > 0.05) between the formulated tablet and the marketed one.

The developed analytical method showed good linearity, accuracy, precision, and specificity. This study recommends a simple, validated analytical method for herbal and food supplement manufacturers to use in quality control of their products.

## 5. Conclusion

In this study, we developed a tablet formulation of rutin 250 mg in our research labs, and we also developed a simple validated UV method for analysis and quantification of rutin in formulated tablets as well as raw material. The dissolution profile of our formulated tablet was slightly more than the marketed rutin tablet. The shelf and accelerated stability study results of the formulated tablet showed that the formulated tablets are stable. This study can guide companies that manufacture herbal products to improve their formulated herbals and apply validated analytical methods to check their product quality.

## Figures and Tables

**Table 1:** Compositions of formulated tablets.

Component	Formula (F1)	Formula (F2)	Formula (F3)
Rutin	250	250	250
MCC	182	180	185
Magnesium stearate	5	5	5
Aerosil	5	5	5
Acdisol	8	10	5

Total weight	450	450	450

**Table 2:** Recovery and intraday precision assay of home prepared formula of rutin 250 mg tablet.

Day	Rutin (mg/ml)			% RSD	ANOVA	% recovery
	Sample number					
	1	2	3			
80%						
1	0.0317	0.0317	0.0313	0.026	0.63	98.55 ± 0.7
2	0.0317	0.0313	0.0317	0.026		98.55 ± 0.7
3	0.0317	0.0320	0.0320	0.026		99.73 ± 0.7

100%						
1	0.0415	0.0415	0.0415	0.000	0.11	103.65 ± 0
2	0.0411	0.0407	0.0407	0.020		102.08 ± 0.5
3	0.0415	0.0411	0.0415	0.020		103.34 ± 0.5

125%						
1	0.0509	0.0509	0.0505	0.016	0.178	101.52 ± 0.4
2	0.0513	0.0513	0.0509	0.016		102.27 ± 0.4
3	0.0509	0.0513	0.0509	0.016		102.02 ± 0.4

**Table 3:** Hardness, diameter, and thickness of the formulated tablet.

Tablet specification	Average	Minimum	Maximum
Weight (mg)	445.4	440.7	450.9
Thickness (mm)	2.31	2.29	2.35
Hardness (N)	253	227	284
Diameter (mm)	13.03	12.99	13.09

**Table 4:** Stability of tablets at room temperature and 40°C.

Day	Tablet stored 40°C (% assay )	Tablet stored at room temperature (% assay)
7	96.38	106.41
14	95.98	103.58
150	91.69	96.33
